# Ca^2+^ imaging and gene expression profiling of *Lonicera Confusa* in response to calcium-rich environment

**DOI:** 10.1038/s41598-018-25611-5

**Published:** 2018-05-04

**Authors:** Wenwen Jin, Yan Long, Chunhua Fu, Libin Zhang, Jun Xiang, Baoshan Wang, Maoteng Li

**Affiliations:** 1 College of Life Science and Technology, Huazhong University of Science and Technology, Wuhan, 430074 China; 2 College of Life Science, Shandong Normal University, Jinan, 250000 China; 3 Hubei Key Laboratory of Economic Forest Germplasm Improvement and Resources Comprehensive Utilization, Hubei Collaborative Innovation Center for the Characteristic Resources Exploitation of Dabie Mountains, Huanggang Normal University, Huanggang, 438000 China; 4 Institute of Biotechnology, Chinese Academy of Agricultural Sciences, Beijing, 100081 China

## Abstract

As a medicinal plant widely planted in southwest karst of China, the study of adaptation mechanisms of *Lonicera confusa*, especially to karst calcium-rich environment, can provide important theoretical basis for repairing desertification by genetic engineering. In this study, the Ca^2+^ imaging in the leaves of *L. confusa* was explored by LSCM (Laser Scanning Confocal Microscopy) and TEM (Transmission Electron Microscopy), which revealed that the calcium could be transported to gland, epidermal hair and stoma in the leaves of *L. confusa* in high-Ca^2+^ environment. In addition, we simulated the growth environment of *L. confusa* and identified DEGs (Differentially Expressed Genes) under different Ca^2+^ concentrations by RNA sequencing. Further analysis showed that these DEGs were assigned with some important biological processes. Furthermore, a complex protein-protein interaction network among DEGs in *L. Confusa* was constructed and some important regulatory genes and transcription factors were identified. Taken together, this study displayed the Ca^2+^ transport and the accumulation of Ca^2+^ channels and pools in *L. Confusa* with high-Ca^2+^ treatment. Moreover, RNA sequencing provided a global picture of differential gene expression patterns in *L. Confusa* with high-Ca^2+^ treatment, which will help to reveal the molecular mechanism of the adaptation of *L. confusa* to high-Ca^2+^ environment in the future.

## Introduction

Currently, ecological deterioration, soil erosion and desertification increase have seriously restricted the development of local economy in karst area of southwestern China. The use of genetic engineering to repair desertification is an important method in vegetation recovery^1^. As one of typical species in southwestern China, the study on the adaptation mechanisms of *Lonicera confusa* to karst environment, especially to karst calcium-rich environment, can provide important theoretical basis for repairing desertification environment by plant genetic engineering.

Calcium ions (Ca^2+^), one of the most abundant metal elements, is available in most soils^2,3^. It is involved in the growth, development and the adaptation to the environment in plants. It is also one of the most important second messenger upon environmental stimulation^4^. Through the cytoplasmic concentration of Ca^2+^ cyclical change, plants response to outside stimulation and produce calcium signals. The calcium target protein, such as calmodulin (CaM), calcium dependence protein kinase (CDPKs) and calcineurin B (CBL), pass down the signal to regulate plant growth, development, photosynthesis and stress resistance^5–7^. On the other side, high concentration of Ca^2+^ in the cell can cause toxicity^8,9^. Excessive Ca^2+^ restricted the growth of many plants in calcareous soils^10^. For instance, high Ca^2+^ interfered with various crucial cell processes, including Ca^2+^ dependent signaling, phosphate-based energy metabolism and microskeletal dynamics^6,11,12^. Previous studies indicated that plant cells have evolved to possess fine mechanism to adjust free Ca^2+^ concentration in the cytoplasm in response to environmental changes, which mainly through the body Ca^2+^ transport system, including Ca^2+^-ATPase and Ca^2+^ channels^8,13–18^. For example, some plant cells can actively transport Ca^2+^ from the cytosol into the vacuole, endoplasmic reticulum, mitochondria, plastids and cell walls^8,13,15,19–21^, and other plants could accumulate crystalline Ca oxalate and deposit it in the storage parenchyma, bundle sheath cell, epidermal trichomes or chlorenchyma^12,22–24^.

*L. confusa* belongs to the family Caprifoliaceae. As one medicinal plant with ecological value^25–33^, *L. confusa* could adapted to karst calcium-rich area of southwest China^34,35^. Our previous results showed that *L. confusa* could excrete excess Ca salts via stomata and store the excess Ca^2+^ in glands and trichomes^34^. However, the underlying molecular mechanisms of *L. confusa* in respond to the excess Ca^2+^ remain to be solved. The research of the adaptability of *L. confusa* in response to the karst calcium-rich environment will benefit to the exploration of theoretical knowledge and ecological restoration.

In this study, the ultrastructure of mature leaves treated with high or low Ca^2+^ was observed by transmission electron microscope (TEM). Moreover, differentially expressed genes (DEGs) are identified under different Ca^2+^concentrations by employing RNA-seq technology and GeneFishing PCR. The results showed that multiple DEGs were related to Ca^2+^ transport and accumulation in *L. confusa*. Furthermore, protein-protein interaction network among DEGs identified some important regulatory genes and transcription factors. In summary, the present results provided the groundwork for revealing the molecular mechanism for the adaptation to calcium-rich environment in *L. confusa*.

## Results

### Calcium in soil could be transported to trichomes, glands, and stoma in the leaves of *L. confusa* with higher Ca^2+^ treatment

The mature leaves of *L. confusa* planted in Nongla Karst Experimental Site (108°19′E, 23°29′N) was performed for microwave digestion and flame atomic absorption spectrometry. The results revealed that the average calcium content was about 5.93 mg · g^−1^, which was much higher than that planted in sandstone soil^36^. In order to identify the distribution law of calcium in the leaves of *L. confusa*, the 0, 25, 50, 75, 100 and 125 mg/L gradient concentration of calcium chloride were used for pouring the plant materials, respectively. The leaves with different concentration of external Ca^2+^ treatment were labeled with Fluo-3/AM ester and examined under LSCM.

**Figure 1:**
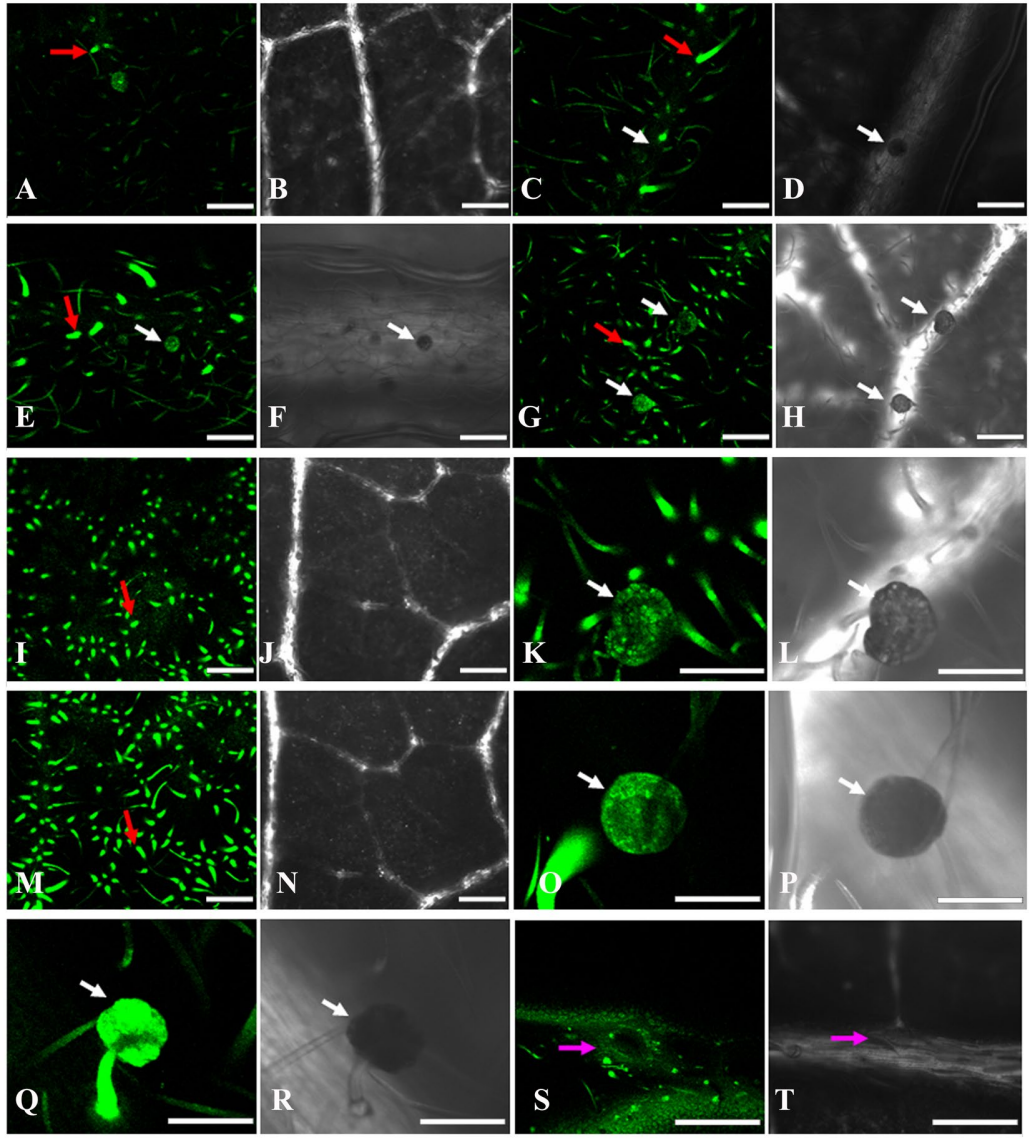
Confocal image and the relative density of the fluorescence of *L. confusa* leaves loaded with Fluo-3/AM. (**A**,**C**,**E**,**G** and **I**) represent the confocal images of *L. confusa* abaxial surface of leaves after 25, 50, 75, 100 and 125 mg/L Ca^2+^ treatment for 36 h. (**B**,**D**,**F**,**H** and **J**) represent the perspective images of (**A**,**C**,**E**,**G** and **J**), respectively; (**K** and **L**) respectively represent the confocal and perspective images of glands after 100 mg/L Ca^2+^ treatment for 36 h; (**M** and **N**) represent the confocal and perspective images of abaxial surface of leaves after 100 mg/L Ca^2+^ treatment for 72 h; (**O** and **Q**) represent the confocal images of glands in leaves after 100 mg/L Ca^2+^ treatment for 72 h; (**P** and **R**) represent the perspective images of glands in leaves after 100 mg/L Ca^2+^ treatment for 72 h; (**S** and **T**) represent the confocal and perspective images of stomata in the leaves after 100 mg/L Ca^2+^ treatment for 72 h. Bar = 100 μm.

The previous studies indicated that Fluo-3 fluorescence intensity is proportional to the changes of Ca^2+^ concentration^37^. The LSCM results in this study revealed that the fluorescent intensity improved with increased external Ca^2+^ treatment (Fig. 1A–J), which indicated that the calcium intake in *L. confusa* and calcium concentration in the soil solution are directly related. Therefore, the leaves in high calcium environment may absorb much more calcium than in normal environment. *L. confusa*, as a calcium resistance plants in karst areas, should possess some mechanisms to avoid excessive Ca^2+^ in the cytoplasm to affect the normal signal transmission and the formation of the cytoskeleton dynamics process. Further observation showed that the fluorescent intensity was mainly distributed in trichomes (Fig. 1, red arrows), glands (Fig. 1, white arrows) and stoma (Fig. 1, pink arrows). This result indicated that the excess of Ca^2+^ absorbed from soil could transport to trichomes, glands, and stoma, which is consistent with our previous results in mature leaves that planted in Nongla Karst Experimental Site^34^. Moreover, the fluorescent intensity further increased with the Ca^2+^ treatment time prolonged (Fig. 1G,K,M,O,Q).

**Figure 2:**
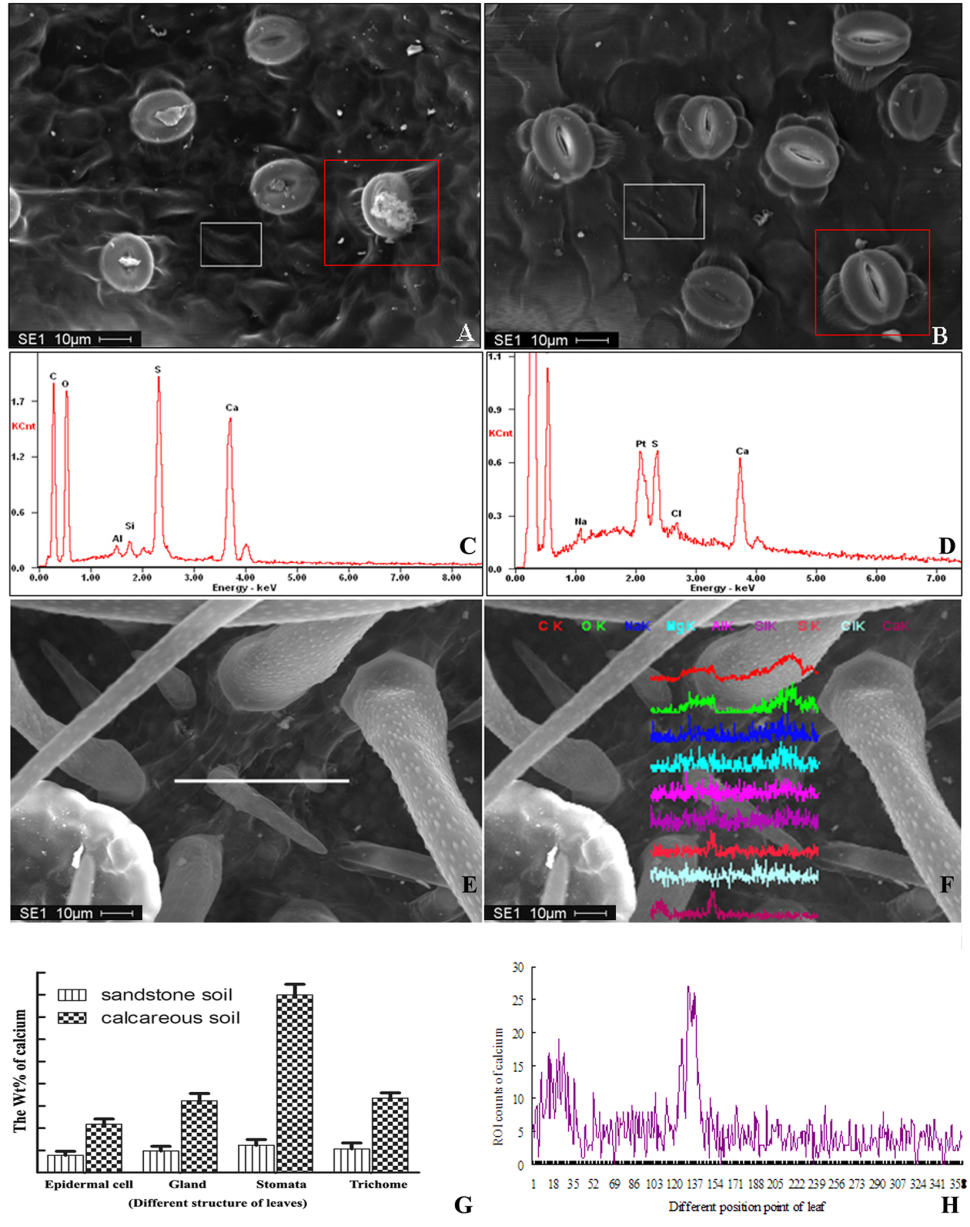
The calcium content analysis by using EDX. (**A** and **B**) represent the SEM (Scanning Electron Microscopy) map of leaves cultivated in calcareous and sandstone soil (white and red square frames represent the map scanning area of epidermal cells, stomata and its surround cells); (**C** and **D**) represent the EDX map of calcium; (**E** and **F**) represent the linear scanning area and the different element change in different scanning point (the different color curve represent different element); (**G**) The Wt % of calcium in different structures cultivated in calcareous and sandstone soil. (**H**) The amplified calcium change curve in different scanning point from (**D**).

The excess of Ca^2+^ could transport to trichomes, glands, and stoma, which was also revealed by using the energy-dispersive X-ray spectrometer for chemical component analysis of the leaves of *L. confusa*. The results showed that the Wt% of calcium in epidermal cell, trichomes, glands, stoma and especially in their surrounding cells of *L. confusa* cultivated in calcareous soil was much higher than that cultivated in sandstone soil (Fig. 2A–D,G). Compared with *L. confusa* in sandstone soil, there were 2.81, 2.68, 2.30 and 6.44-fold increasement of Wt% in epidermal cell, trichomes, glands, stoma and their surrounding cells of *L. confusa* in calcareous soil. The content of calcium in the different leaf structure was also measured by using linear scanning method and we found that the ROT count of calcium in trichomes part was much higher than that of other parts (Fig. 2F,H). These results again indicated the excess of Ca^2+^ absorbed from calcium rich environment could transport to trichomes, glands, and stoma.

### Many calcium channels and pools are found in the leaves of *L. confusa* in high calcium environment

**Figure 3:**
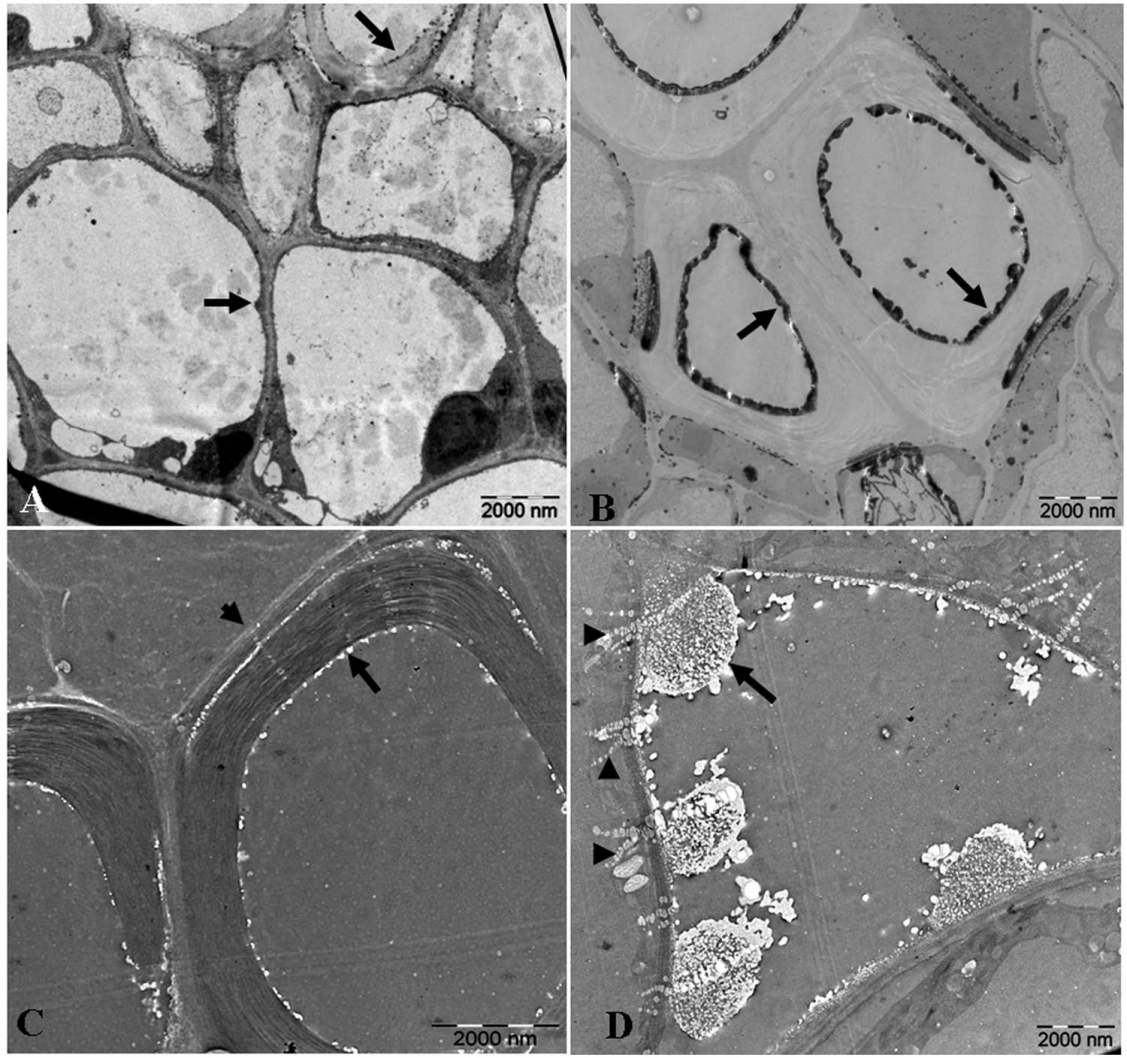
TEM analysis of *L. confusa* leaves with high and low Ca^2+^ treatment. (**A** and **B**) represent TEM analysis of antimonite-labeled cells located in white square frames in Fig. 2b (low Ca^2+^ treatment, 25 mg/L) and Fig. 2a (high Ca^2+^ treatment, 125 mg/L), respectively (Arrow indicates the calcium antimonate precipitate), (**C** and **D**) represent the TEM analysis of antimonite-labeled cells near to glands, stoma in low and high Ca^2+^ treatment leaves, respectively. The antimonite-labeled cells were treated with EGTA and the calcium antimonate precipitates were chelated (Arrows indicate the calcium pools and arrow heads indicate the calcium channels).

As mentioned above, the calcium content in leaves of *L. confusa* planted in calcium rich soil was higher than that in calcium poor soil. In order to make clear how the calcium distributed in cells of *L. confusa*, potassium antimonate was used to localize the calcium in cells by using TEM technique. The smaller and fewer calcium antimonate precipitate were observed in the cells of in *L. confusa* treated with lower concentration of Ca^2+^ (25 mg/L, Fig. 3A). In contrast, the relatively larger and orderly arranged stored calcium were observed along the cell wall in the materials treated with higher level of Ca^2+^ (125 mg/L, Fig. 3B). This result indicated that formation of the common crystalline formation (such as calcium oxalate) arranged across the cell wall was another important mechanism for *L. confusa* to adapt the higher Ca^2+^ environment except for three other mechanisms we reported before^34^.

Calcium antimonate can be chelated by EGTA. Therefore, the antimonite-labeled cells near to glands, stoma in low and high Ca^2+^ treatment leaves were treated with EGTA. TEM results showed that there are obviously more Ca^2+^ pools in the leaves of *L. confusa* treated with higher Ca^2+^ for a certain time (Fig. 3D), and fewer Ca^2+^ pools in lower Ca^2+^ treated materials (Fig. 3C). Very interestingly, we also observed that more calcium ion channels in the *L. confusa* treated with higher Ca^2+^ than in lower Ca^2+^ treated materials (Fig. 3C,D, arrow heads), which suggested that *L. confusa* could store the Ca^2+^ in the calcium pools. Taken together, our results indicated more calcium channels and pools were distributed in the leaves of *L. confusa* in high-Ca^2+^ environment than in low-Ca^2+^ environment.

### Identification of differentially expressed genes by RNA sequencing in higher and lower calcium-treated *L. confusa*

**Figure 4:**
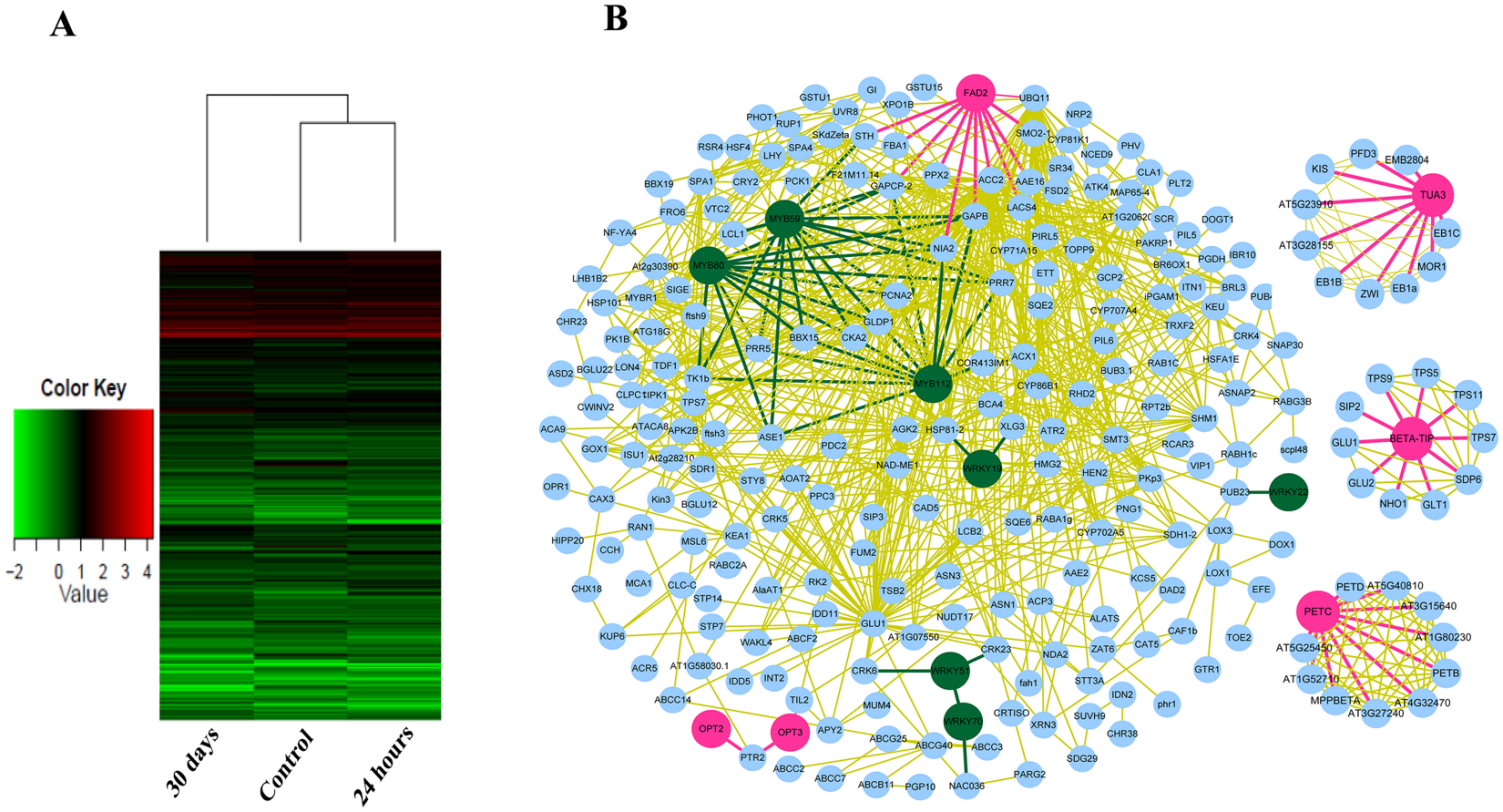
(**A**) Heatmap analysis of differentially expressed genes among control (no Ca^2+^ treatment), 24-hour Ca^2+^ treatment and 30-day Ca^2+^ treatment *L. Confusa*. (**B**) Protein-protein interaction network of differentially expressed genes in *L. Confusa* constructed by Cytoscape software. Red circles indicate the differentially expressed genes identified in Genefishing experiments. Green circles indicate the identified transcription factors.

The *L. confusa* treated with 125 mg/L calcium chloride solution and pure water (as control) for 24 hours and 30 days were used for DEG analysis. Two biological replicates of RNA-seq of *L. confusa* leaf tissues treated with different level of Ca^2+^ were performed. The two sets of the corresponding sequencing samples were combined, which generated 57393938, 69001767 and 60782634 clean reads, respectively. The obtained clean reads were then mapped to the assembled transcriptome of *L. confusa*^38^. The results showed that 35584241, 44851148 and 38596972 clean reads can be mapped to the assembled unigenes^38^, respectively. Among the mapped reads, 24908968, 32292826 and 28171538 clean reads were uniquely mapped to the unigenes, respectively. 10675273, 12558322 and 10425434 clean reads were mapped to multiple locations of unigenes, respectively. After mapping, 94607, 90574 and 86396 expressed unigenes for 0 h, 24-hour and 30-day Ca^2+^-treatment were obtained, respectively. To identify the DEGs between control and calcium-treated *L. Confusa*, we set the expression level of unigenes of control as a control and investigated the up- or down-regulated unigenes of calcium-treated *L. confusa*. The analysis showed that 322,69 unigenes were differentially expressed between control and 24-hour calcium-treatment *L. confusa*, and 43,148 unigenes were differentially expressed between control and 30-day calcium-treatment *L. confusa*. Also, we observed that a total of 34,155 unigenes were differentially expressed between 24-hour and 30-day calcium-treatment *L. confusa* samples, which indicated that short-term (24-hour) and long-term (30-day) Ca^2+^ treatment to *L. confusa* may induce different physiological status of *L. confusa*. As shown in Supplementary Fig. 1, these DEGs between control and calcium-treated *L. Confusa* were further annotated with GO terms. Furthermore, the GO-enriched DEGs between control and calcium-treated *L. Confusa* were displayed by heatmap analysis (Fig. 4A). We next performed GeneFishing PCR to validate the DEGs identified in RNA sequencing analysis. The *L. confusa* treated with 125 mg/L calcium chloride solution and pure water (as control) for 30 days were used for GeneFishing analysis. In all, 24 DEGs were identified between control and high Ca^2+^-treated *L. confusa* (Supplementary Table 1). The DEGs were further confirmed by semi-quantitative RT-PCR experiments (Supplementary Fig. 2), which was very consistent with the RNA sequencing results.

Furthermore, Cytoscape software^39^ was used to construct a complex protein-protein interaction network among DEGs in *L. confusa*. As shown in Fig. 4B, there were 253 nodes and 762 edges obtained in the network. Some important transcription factors, such as MYB59, WRKY19, WRKY51 and WRKY70 were involved in the network. Also, Tua3 (alpha-3 tubulin), one of the important cytoskeleton components, interacts with 9 targets including EB1C, EB1A, EB1B, ZWI, EMB2804, KIS and so on, in which all target proteins are associated with the formation and biological functions of microtubule. Therefore, the results indicate that Tua3 and target proteins may form a complex network to regulate cell morphology and intercellular transportation *L. confusa*.

### Functional Enrichment of differentially expressed genes

**Figure 5:**
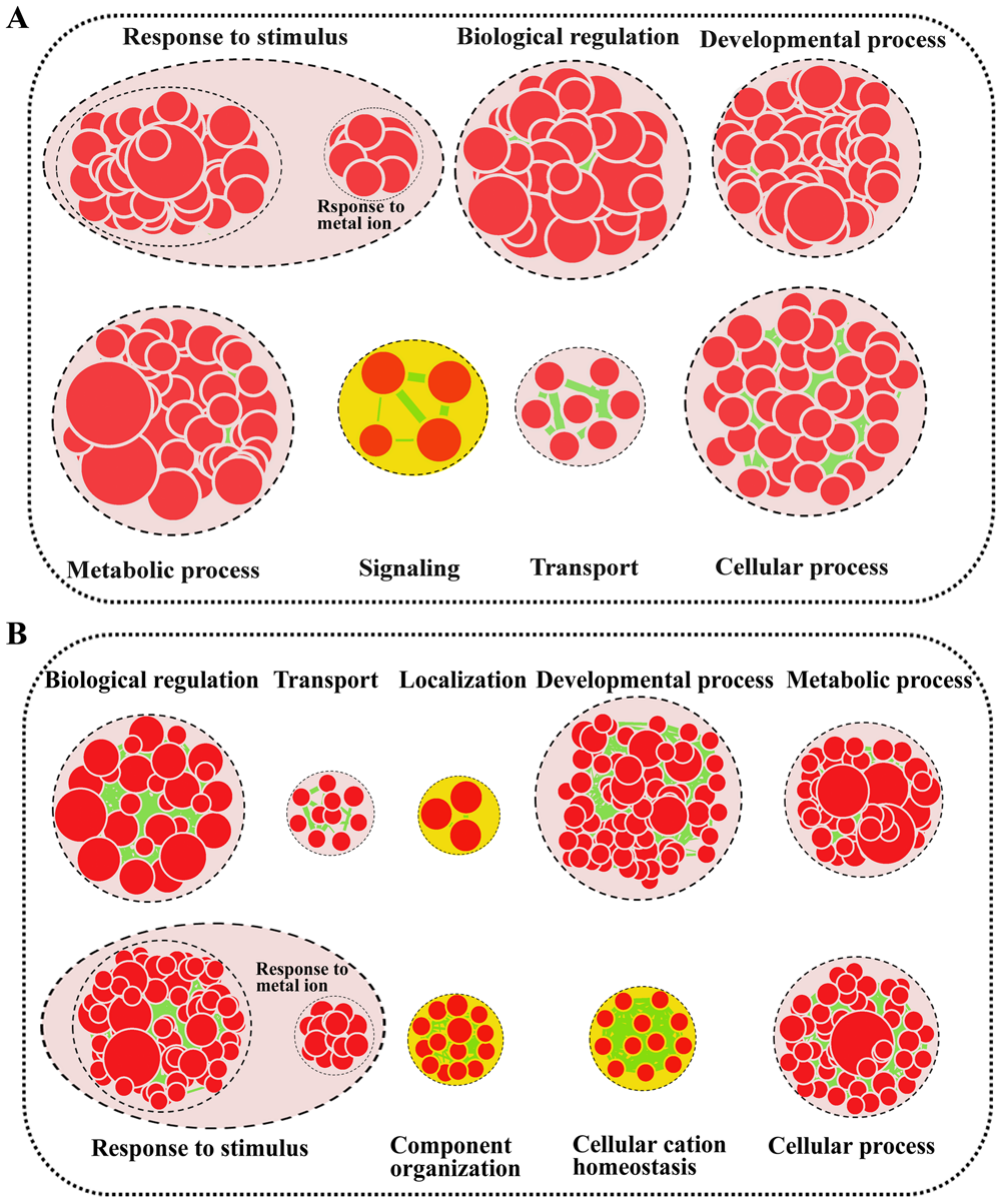
Biological Process analysis of differentially expressed genes (DEGs) between short-term (24 hours) Ca^2+^ -treated and long-term Ca^2+^ treated *L. confusa*. GO modules enriched with short-term (24 hours) Ca^2+^ -treated DEGs (**A**) and long-term (30 days) Ca^2+^ treated DEGs (**B**) were visualized by the EnrichmentMap in Cytoscape. The red and yellow circles indicate the common and different biological processes between short-term (24 hours) Ca^2+^ -treated and long-term Ca^2+^ -treated *L. confusa*, respectively.

For functional annotation of DEGs of short-term and long-term Ca^2+^ induction *L. confusa*, the DEGs were further analyzed using Cytoscape EnrichmentMap (http://www.cytoscape.org/). As shown in Fig. 5, EnrichmentMap analysis of short-term and long-term Ca^2+^ induction DEGs generated 300 and 404 nodes, respectively. These nodes were classified into different categories. The common terms between short-term (24 hours) (Fig. 5A) and long-term (30 days) (Fig. 5B) Ca^2+^ induction DEGs were “Response to stimulus”, “Developmental process”, “Biological regulation”, “Metabolic process”, “Cellular process” and “Transport”. Very importantly, some DEGs were enriched in different categories. For example, EnrichmentMap analysis showed that short-term Ca^2+^ induction DEGs were also clustered in “Signaling”. This result indicated that short-term Ca^2+^ induction in *L. confusa* might activate some important signal transduction pathways and/or defense responses. Nevertheless, long-term Ca^2+^ induction DEGs were mainly clustered in “Cellular cation homeostasis”, “Component organization” and “Localization”. Interestingly, the enrichment of “Cellular cation homeostasis” suggested that *L. confusa* gradually adapted to high- Ca^2+^ environment and kept a new cation balance after long-term Ca^2+^ treatment. Meanwhile, “Transport” process was enriched both in short-term and long-term Ca^2+^ induction *L. Confusa*, which suggested that Ca^2+^ treatment induced the activation of relative Ca^2+^ transport pathways in *L. Confusa*.

### KEGG pathway analysis

To reveal biological functions of the identified genes, the DEGs between control (no Ca^2+^ treatment) and 30-day Ca^2+^ treatment *L. Confusa* were further assigned for KEGG pathway analysis. As shown in Supplementary Dataset 1, a total of 99 KEGG pathways were ranked by p-value. The top 5 pathways included “Ribosome” (91 unigenes, P = 3.97 × e^−7^), “Phenylpropanoid biosynthesis” (61 unigenes, P = 1.74 × e^−6^), “Methane metabolism” (P = 2.39 × e^−6^), “Cyanoamino acid metabolism” (29 unigenes, P = 3.46 × e^−5^) and “Phenylalanine metabolism” (50 unigenes, P = 7.06 × e^−5^). Furthermore, we extracted the Ca^2+^ metabolism-related pathways for analysis. As shown in Supplementary Fig. 3, the Ca^2+^ metabolism-related pathways were classified into five categories including Cellular Processes, Environmental Information Processing, Genetic Information Processing, Metabolism and Organismal Systems. Among them, Cellular Processes category enriched 142 genes in which 32 genes are involved in the pathway of “Transport and catabolism”. More importantly, we observed that a lot of (265) genes are involved in “Signal transduction” pathway in Environmental Information Processing category. These results indicated long-term Ca^2+^ treatment on *L. Confusa* may induce Ca^2+^ transport and related signal transduction processes.

## Discussion

*L.confusa* is a woody perennial, evergreen and twining vine. It is widely cultivated in eastern Asia as an important medicinal plant. *L.confusa* is also processed into food and a healthy beverage, which facilitates the rapid increase of its commercial value in herbal medicine markets. However, the economy development has been greatly impeded by ecological deterioration, soil erosion and desertification in karst area of southwestern China. *L.confusa* is one of typical species in southwestern China. Therefore, this study focused on the adaptation mechanism of *L. confusa* to karst calcium-rich environment, which will reveal important theoretical basis for repairing desertification environment.

Various studies have revealed that Ca^2+^ will accumulate to a very high level over time in cells when water evaporates from the surface of plants. Moreover, the excess Ca^2+^ can be precipitated as inactive Ca oxalate^22^. Within plants, most long-distance Ca^2+^ transports through plant tissues have been demonstrated to follow apoplastic pathways^40,41^. After the Ca^2+^ transfer to the plant cell, Ca^2+^ can be transported from the cytosol into the vacuole, endoplasmic reticulum, mitochondria, plastids and cell walls where Ca^2+^ can be captured by H^+^/Ca^2+^ antiporters (CAX) and Ca^2+^-ATPases (ACA)^8,21^. CAX1 has a primary role in Ca^2+^ accumulation in the leaf mesophyll and it is important in controlling apoplastic Ca^2+^, stomatal aperture, and growth^41,42^. In this study, we also observed high-Ca^2+^ treatment induced the transport of Ca^2+^ to trichomes, glands, and stoma in the leaves of *L. Confusa*. Moreover, more calcium channels and calcium pool are accumulated in the leaves of *L. confusa* in high calcium environment. Although the signaling role of Ca^2+^ in organisms has developed rapidly^3,21^, fundamental information regarding the mechanisms that regulate Ca^2+^ transport and storage in plants still remains elusive^43^.

RNA-seq technology is an effective transcriptome analysis tool, which can detect novel and rare RNA transcripts and accurately quantify gene expression level. In this study, we identified multiple DEGs (differentially expressed genes) between control and calcium-treated *L. Confusa*. Bioinformatics analysis showed that 322,69 unigenes were differentially expressed between control and 24-hour calcium-treatment *L. confusa*, and 43,148 unigenes were differentially expressed between control and 30-day calcium-treatment *L. confusa*. Importantly, the different gene expression level between 0-hour and 24-hour or 30-day Ca^2+^-treated *L. confusa* might be induced not only by the Ca^2+^ treatment but also by own natural growth of *L. confusa*. Therefore, we could not fully exclude the effect of 24-hour or 30-day natural growth of *L. confusa* on the gene expression level. Nevertheless, the samples used for RNA-seq were mature leaves of *L. confuse*. We supposed that the gene expression level in the mature leaves is very stable in 24 hours or 30 days, and the different gene expression level between 0-hour and 24-hour or 30-day Ca^2+^-treated *L. confusa* were mainly induced by Ca^2+^ treatment. Furthermore, the identified DEGs were validated by GeneFishing PCR. For example, Tua3, CaM, CDPKs, CBL and some other important genes were identified. Tua3 is one of the important cytoskeleton components. Our network analysis showed that Tua3 interacts with 9 target genes, in which all target genes are associated with the formation of microtubule. Taken together, these results indicate that Tua3 and target genes may regulate cell morphology and intercellular transportation by a complex gene interaction network in *L. confusa*. In the meanwhile, it has been reported that CaM, CDPKs and CBL are involved in cytoskeleton formation and movement, osmotic stress resistance^44,45^. Calcium, as the second messenger upon environmental stimulation in plant, plays critical roles in response to outside signals and activates the expression of related genes. For Ca^2+^ transport into the vacuole in plant cells, both Ca^2+^-ATPases (ACA) and Ca^2+^/H^+^ antiporters (CAX) play important roles^46^. Therefore, transcript abundance of transporters could be up-regulated under conditions of adequate Ca supply. Also, proteins that modify activity of transporters, such as CAM or CXIP, could also be regulated in a similar fashion^47,48^. For instance, compared with epidermal cells, mesophyll cells of *Arabidopsis* have the higher capacity to store Ca^2+^ in their vacuoles by virtue of the higher expression of the Ca^2+^/H^+^ antiporter on the tonoplast membrane^41^. Our DEGs analysis in this study showed that CaM and CAX were upregulated in long-term Ca^2+^ treated *L. Confusa*, which might reflect the adaptive changes of *L. Confusa* to high-Ca^2+^ environment. In addition, EnrichmentMap analysis showed that multiple DEGs were involved in “Cellular cation homeostais” and “Transport” processes. The results suggested that Ca^2+^ treatment induced the activation of relative Ca^2+^ transport pathways and *L. confusa* gradually adapted to high-Ca^2+^ environment and kept a new cation balance after long-term Ca^2+^ treatment, which is very consistent with our LSCM and TEM results. Collectively, identification of these important DEGs will help us to explore the possible regulatory mechanism of the adaptation to high-Ca^2+^ environment in *L. confusa*.

## Materials and Methods

### Plant materials

*L. confusa* cultivars were taken from typical Nongla Karst Experimental Site (108°19′E, 23°29′N) as previously described^34^. The *L. confusa* cultivars were planted in the soils transported from Nongla Karst Experimental Site. In order to remove the calcium ions in the soils, the soils were washed with pure water for several times. Furthermore, 24 basin of *L. confusa* materials were divided into six groups and treated with different concentrations of Ca^2+^, respectively.

### Detection of Ca^2+^ on the leaves of *L. confusa* by LSCM

The Ca^2+^ images in the leaves of *L. confusa* were detected as previously described^34,49^. The *L. confusa* leaves growing in soils with different level Ca^2+^ were soaked in Fluo-3/AM ester solution at 4 °C for 2 h. These *L. confusa* leaves were then washed with PBS solutions and observed under Olympus FV1000 LSCM (OLYMPUS, JAPAN).

### TEM analysis

TEM analysis of the leaves of *L. confusa* was performed as previously described^34,50^ except for small modification. First, 5 independent leaf samples of *L. confusa* with different level Ca^2+^ treatment were isolated, washed with phosphate buffer solution and cut into 0.2 cm × 0.2 cm slices. Furthermore, these samples were fixed and dehydrated through graded ethanol concentrations once for 10 min and soaked in 100% acetone twice for 30 min. Subsequently, the samples were treated with 2% potassium pyroantimonate and dehydrated. Finally, the samples were embedded with Spurr epoxy resin and sectioned with superfine section machine. The obtained sections were dyed and examined using TEM (Dutch FEI Tecnai F20-Twin, Netherlands).

### RNA isolation and GeneFishing PCR

The total RNA isolation of the *L. confusa* leaves treated with different level of Ca^2+^ was performed as previously described^51^. GeneFishing kit (Seegene, Inc.) was used for differential display PCR analysis. GeneFishing^TM^ PCR were done with dT-ACP2 and 20 pairs of random primers, the PCR products were run on 2% agarose gel and the cDNA bands ranged from 100 and 1.5 kb were used for cloning and sequencing (Supplementary Fig. 2). QIAquick Gel extraction kit (Qiagen) was used to isolate the differentially expressed bands. The isolated DNA fragments were cloned into pGEM^®^-T Easy vector (Promega) and sequenced. Further Semi-quantitative RT- PCR experiments were utilized for the validation of the GeneFishing results. The Primers used for GeneFishing PCR and Semi-quantitative RT- PCR were shown in Supplementary Tables 2 and 3, respectively.

### RNA sequencing and DEG analysis

The RNA-seq cDNA libraries of *L. confusa* leaves treated with different level of Ca^2+^ were constructed using TruSeqTM RNA Sample Preparation Kit (Illumina, Inc.). Shortly, oligo (dT) magnetic beads (NEB) were used to capture poly-A mRNAs from the isolated total RNA. The captured mRNAs were fragmented into 200–500 bp for cDNA synthesis and adaptor ligations. The synthesized cDNAs were PCR-amplified, quantified and sequenced on Illumina HiSeq 2000 using 2 × 100 bp pair-end sequencing protocol. The generated clean reads were deposited in NCBI Sequence Read Archive (SRA) Sequence Database (Accession number: SRR6024635). Bioinformatics analysis of differentially expressed genes of RNA-seq samples was performed as described^37^. Briefly, gene expression level of each RNA-seq sample was measured by RPKM (Reads Per kb per Million reads). The genes with RPKM ≥1 were regarded to be expressed in the RNA-seq analysis. The edgeR software was then used to identify the differentially expressed genes. The differentially expressed genes among L. confusa leaves treated with different level of Ca^2+^ were selected using a log FC (log-fold expression change) >2 or <−2, a false discovery rate (FDR < 0.001) and p-value < 0.005 as the threshold value.

### Pathway analysis and interaction analysis

The functions of the DEGs were analyzed by using Kyoto Encyclopedia of Genes and Genomes (KEGG) database (http://www.genome.jp/tools/blast). Cytoscape_2_6_3 (http://www.cytoscape.org/plugins/index.php) was used to analyze the protein-protein interaction among DEGs.

### Accession number

RNA sequencing data have been deposited in NCBI Sequence Read Archive (SRA) Sequence Database with accession number SRR6024635.

## Electronic supplementary material

Supplementary information

Dataset 1
