# Supplementary material for: Ca2+ imaging and gene expression profiling of Lonicera Confusa in response to calcium-rich environment
Source: Sci Rep. 2018 May 4;8:7068. doi: 10.1038/s41598-018-25611-5 (PMC5935734; doi:10.1038/s41598-018-25611-5)
Supplement: Supplementary file 1 — Supplementary information [file 41598_2018_25611_MOESM1_ESM.pdf]

Ca^{2+} imaging and gene expression profiling of *Lonicera Confusa* in response to calcium-rich environment

Wenwen Jin[1,3], Yan Long[4], Chunhua Fu[1], Libin Zhang[1*], Jun Xiang[3*], Baoshan Wang[2], Maoteng Li[1,3]

[1]College of Life Science and Technology, Huazhong University of Science and Technology, Wuhan 430074 China;

[2]College of Life Science, Shandong Normal University, Jinan, 250000, China;

[3]Hubei Key Laboratory of Economic Forest Germplasm Improvement and Resources Comprehensive Utilization, Hubei Collaborative Innovation Center for the Characteristic Resources Exploitation of Dabie Mountains, Huanggang Normal University, Huanggang, 438000, China;

[4]Institute of Biotechnology, Chinese Academy of Agricultural Sciences, Beijing, 100081 China.

*Corresponding author: libinzhang@hust.edu.cn; swxj@hgnu.edu.cn

Supplementary Information: This file contains 4 supplementary figures and 3 supplementary tables.

Figure legends:

Supplementary Fig 1. Gene ontology analysis of DEGs between control and 125mg/L Ca^{2+}-treated (24 hours and 30 days, respectively) *L. Confusa* samples. Green, red and blue bars indicate down-regulated, up-regulated and total DEGs numbers, respectively. (A). Gene ontology analysis of DEGs between control and 125mg/L Ca^{2+}-treated (30 days) *L. Confusa* samples. (B). Gene ontology analysis of DEGs between control and 125mg/L Ca^{2+}-treated (24 hours) *L. Confusa* samples. (C). Gene ontology analysis of DEGs between 125mg/L Ca^{2+}-treated (24 hours) and 125mg/L Ca^{2+}-treated (30 days) *L. Confusa* samples.

Supplementary Fig 2. GeneFishing analysis of higher calcium treated materials and RT-PCR analysis of DEGs. The calcium treatment time was 30 days. Left panel： represents the GeneFishingTM results between 125 mg/L calcium treated samples and control (1 and 2 represent control and 125 mg/L calcium treated samples, respectively; GP represent GeneFishing primers); Right panel: RT-PCR analysis of different DEGs. Randomly selected DEGs were validated between control (0) and different Ca^{2+} (25, 50, 75, 100, 125 mg/L)-treated *L. confusa* using semi-quantitative RT-PCR experiments.

Supplementary Fig 3. The KEGG classification analysis of Ca^{2+}-related DEGs between control and 125mg/L Ca^{2+}-treated *L. Confusa* samples. The Ca^{2+}-related DEGs were classified into 5 categories including Metabolism, Genetic information processing, Environmental information processing, Cellular processes and Organismal systems.

Supplementary Fig 4. The original scans of the gels in Supplementary Figure 2. (A).

The original gel scan of DEG4 RT-PCR analysis. (B). The original gel scan of DEG9 RT-PCR analysis. (C). The original gel scan of DEG10 RT-PCR analysis. (D). The original gel scan of DEG12 RT-PCR analysis. (E). The original gel scan of DEG15 RT-PCR analysis. (F). The original gel scan of 18S rRNA RT-PCR analysis.

Supplementary Fig 1.

A

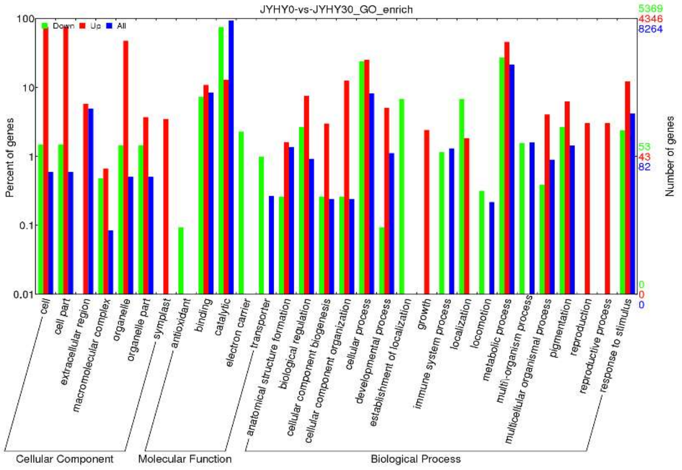

B

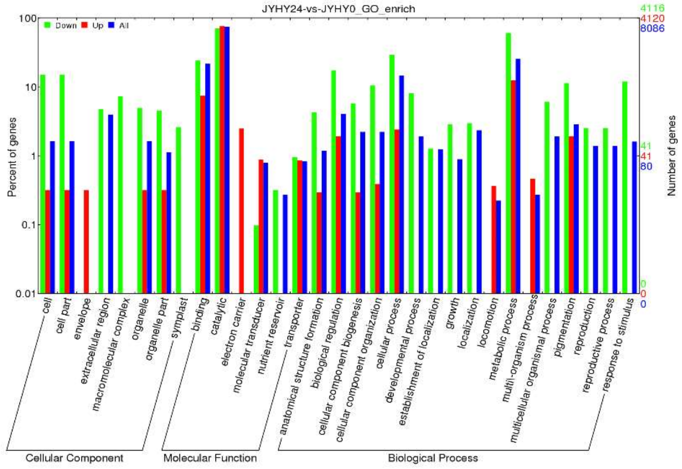

C

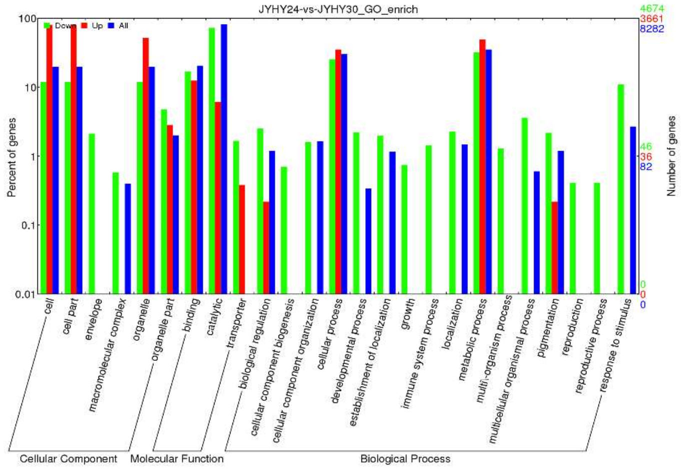

Supplementary Fig 2.

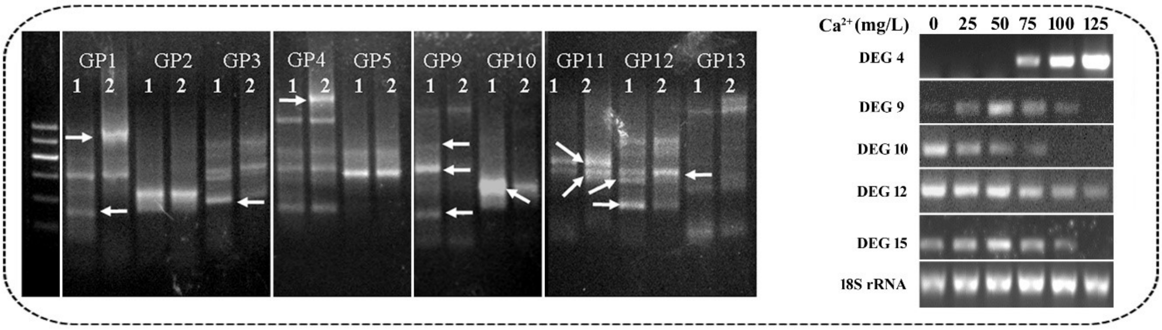

Supplementary Fig 3.

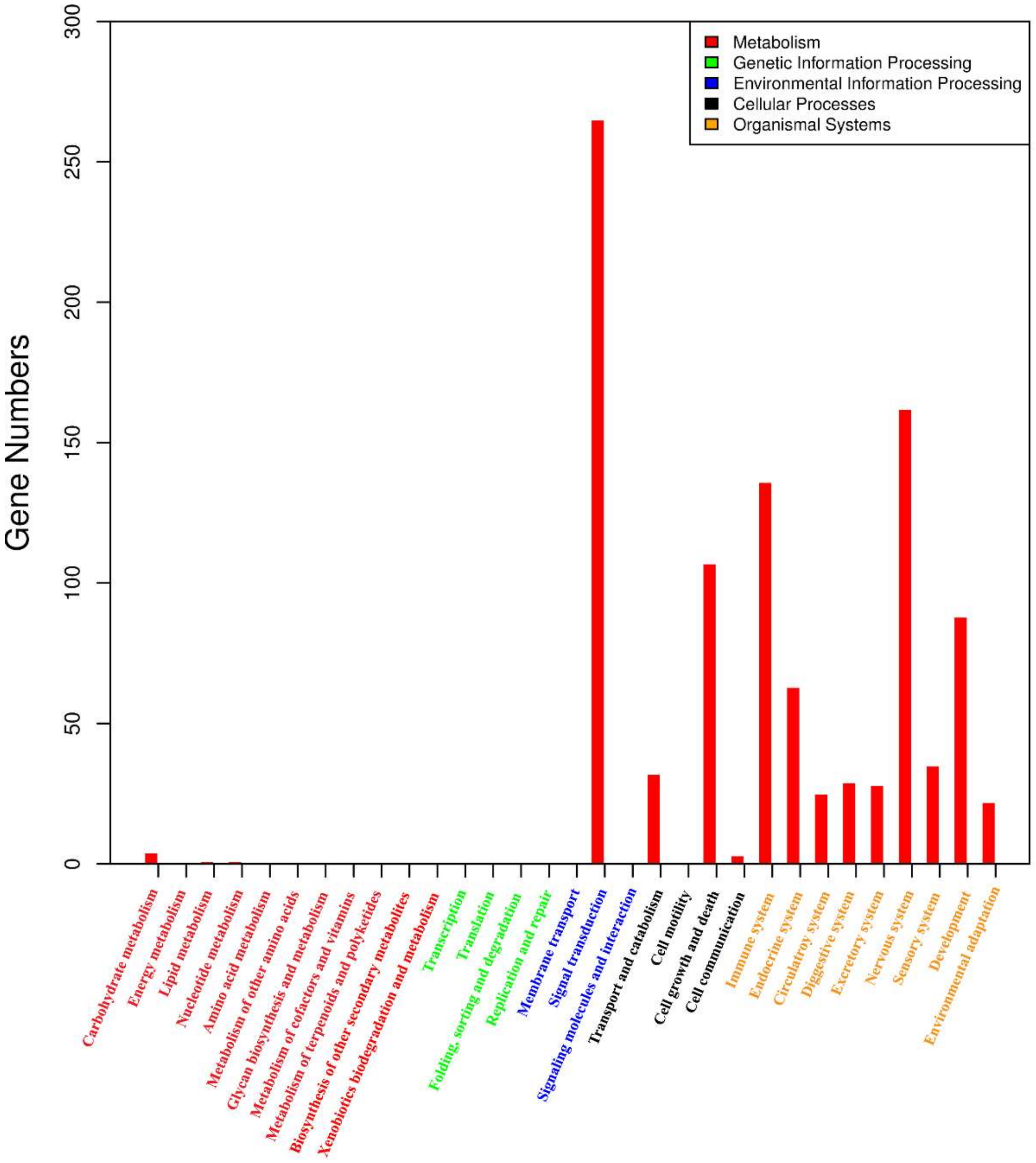

Supplementary Fig 4.

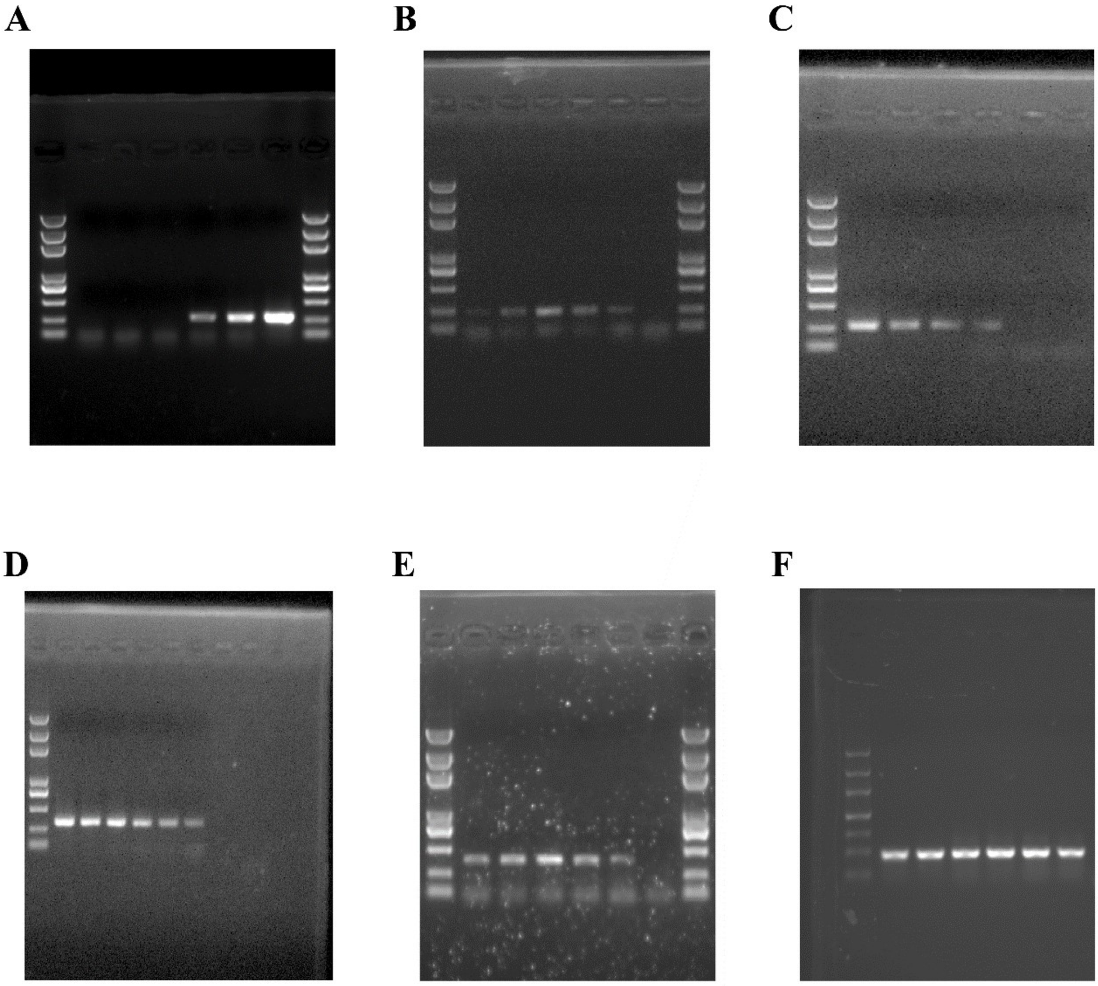

Supplementary Tables

Supplementary Table 1: Blastn analysis of DEGs

DEG	Accession	Description	Max score	E value	Max ident
1	DQ237285.1	*Panax ginseng* tonoplast intrinsic protein (TIP) mRNA, complete cds	212	2e-51	78%
2		unknown			
3	HM773394.1	*Musa* AB Group rubisco activase (Rca) mRNA, partial cds	138	2e-29	80%
4	XM_002302126.1	*Populus trichocarpa* predicted protein, mRNA	307	4e-80	72%
5	XM_002519612.1	*Ricinus communis* (S)-2-hydroxy-acid oxidase, putative	188	1e-44	82%
6	EU872254.1	*Portulaca oleracea* omega-6 desaturase (FAD2) mRNA, partial cds	432	7e-115	82%
7		Unknown			
8		Unknown			
9	gb|AF022389.1|AF022389	*Vigna unguiculata* ADP-ribosylation factor (ARF) mRNA, complete cds	616	2e-172	89%
10	AM392362.1	*Plantago* major partial mRNA for alpha-2 tubulin (TUA2 gene),	453	4e-124	87%
11	CU228904.1	Populus EST from severe droulht-stressed leaves	48.2	0.015	78%
12	ref|XM_002513985.1|	*Ricinus communis* AP-4 complex subunit mu-1	333	1e-87	82%
13	XM_002264518.1	PREDICTED: Vitis vinifera hypothetical protein LOC100245386 (LOC100245386), mRNA	439	1e-119	76%
14		Unknown			

15	XM_002524586.1	*Ricinus communis* chaperone binding protein, putative, mRNA	147	6e-32	77%
16	AY032674.1	*Nicotiana tabacum* peroxidase (PER9-6) mRNA, complete cds	269	1e-68	70%
17	L36680.1	*Pisum sativum* S-adenosylmethionine synthase mRNA	322	1e-84	86%
18	AY059007.1	*Maticora bivirgata* NADH dehydrogenase subunit 2 gene, complete cds; mitochondrial gene for mitochondrial product	41.0	2.8	83%
19	XM_002528766.1	*Ricinus communis* oligopeptide transporter (OPT), putative, mRNA	102	2e-18	73%
20	AY622990.1	*Lycopersicon esculentum* UDP-glucose:protein transglucosylase-like protein SlUPTG1 mRNA, complete cds	66.2	1e-07	88%
21	XM_002521115.1	*Ricinus communis* photosystem I reaction center subunit IV A, chloroplast precursor, mRNA	134	2e-28	88%
22	XM_002509540.1	*Ricinus communis* conserved hypothetical protein, mRNA	405	3e-109	75%
23	NM_178964.2	*Arabidopsis thaliana* PETC（Photosynthetic Electron Transfer C）	266	2e-67	79%
24	XM_002534085.1	*Ricinus communis* Thioredoxin H-type (TH), mRNA	141	1e-30	75%

Supplementary Table 2: Primers used for GeneFishing PCR

Primer Name	Primer sequence
dTACP1	5'-CTGTGAATGCTGCGACTACGATIIIII(T)$_{18}$-3'
ACP1	5'-GTCTACCAGGCATTCGCTTCATIIIIIGCCATCGACC-3'
ACP2	5'-GTCTACCAGGCATTCGCTTCATIIIIIAGGCGATGCC-3'
ACP3	5'-GTCTACCAGGCATTCGCTTCATIIIIICCGGAGGATG-3'
ACP4	5'-GTCTACCAGGCATTCGCTTCATIIIIIGCTGCTCGCG-3'
ACP5	5'-GTCTACCAGGCATTCGCTTCATIIIIIAGTGCGCGCG-3'
ACP6	5'-GTCTACCAGGCATTCGCTTCATIIIIIGGCCACATCG-3'
ACP7	5'-GTCTACCAGGCATTCGCTTCATIIIIICTGCGGATCG-3'
ACP8	5'-GTCTACCAGGCATTCGCTTCATIIIIIGATGCCGCTG-3'
ACP9	5'-GTCTACCAGGCATTCGCTTCATIIIIIGATGCCGCTG-3'
ACP10	5'-GTCTACCAGGCATTCGCTTCATIIIIITGGTCGTGCC-3'
ACP11	5'-GTCTACCAGGCATTCGCTTCATIIIIICTGCAGGACC-3'
ACP12	5'-GTCTACCAGGCATTCGCTTCATIIIIIACCGTGGACG-3'
ACP13	5'-GTCTACCAGGCATTCGCTTCATIIIIIGCAAGTCGGC-3'
ACP14	5'-GTCTACCAGGCATTCGCTTCATIIIIIGCAAGTCGGC-3'
ACP15	5'-GTCTACCAGGCATTCGCTTCATIIIIICCACCGTGTG-3'
ACP16	5'-GTCTACCAGGCATTCGCTTCATIIIIIGTGGACGGTG-3'
ACP17	5'-GTCTACCAGGCATTCGCTTCATIIIIICAAGCCCACG-3'
ACP18	5'-GTCTACCAGGCATTCGCTTCATIIIIICGGAGCATCC-3'
ACP19	5'-GTCTACCAGGCATTCGCTTCATIIIIICTCTGCGAGC-3'
ACP20	5'-GTCTACCAGGCATTCGCTTCATIIIIIGACGTTGGCG-3'
dTACP2	5'-CTGTGAATGCTGCGACTACGATIIIII(T)$_{15}$-3'

Supplementary Table 3：Primers used for DEGs PCR

DEG No.	Forward primer sequence (5’-3’)	Reverse primer sequence (5’-3’)
1	GGCGAAATCGGAATCATC	AGATGAGCTCGTACACAG
3	TGCCAACAAGGATGCTAT	ACGATGAAGCGTCAAACA
4	GGACCGGACTATGATGTTCCT	CCGAATTTCTCACCGAGCAG
5	CACATCGGGAAATCAAAG	GGTCACAACATGCAACAG
6	GCGGACTTGTATGGATTT	TCCCTGCTTCTACATTTT
9	ATGCCGCTGTTGCCACTA	CAATAATCTTCACCCTCCT
10	GAGTGACGTATCTGACCCAT	TGGAACCTGGAGCTGTTG
12	TTCACAGTTTGGGATGTT	AAGCCAATCCAGCCCTTC
14	GCAGAAGCCGAAGTAGCAA	AGTTATCATCAACCCCATT
15	CTACCCAGTTGTTGTGAG	ACTTGCTATTTAGCAGGA
17	CTTATGCCATTGGTGTTC	ATTGTCCGCTAGGAGTTA
21	GGTGTTTTCATTGGCGGC	CGTCCCAATCAGTGGCAAT
22	AGGACCGTTTGTTTGTTA	CTTACTGTTCTATTCCCTTT
23	CCTTCGCTGCCTCACTAT	TACACTCCTTTGCCTCCC
24	CGCTCCATTCCTCGCAGAGTT	AGAACTTGTGAACCTTTA
18S rRNA	GTAACAAGGTTTCCGTAGGTG	ACCACCACTCGTCGTGACGT
